# Burn-induced muscle metabolic derangements and mitochondrial dysfunction are associated with activation of HIF-1α and mTORC1: Role of protein farnesylation

**DOI:** 10.1038/s41598-017-07011-3

**Published:** 2017-07-26

**Authors:** Harumasa Nakazawa, Kazuhiro Ikeda, Shohei Shinozaki, Masayuki Kobayashi, Yuichi Ikegami, Ming Fu, Tomoyuki Nakamura, Shingo Yasuhara, Yong-Ming Yu, J. A. Jeevendra Martyn, Ronald G. Tompkins, Kentaro Shimokado, Tomoko Yorozu, Hideki Ito, Satoshi Inoue, Masao Kaneki

**Affiliations:** 1 Department of Anesthesia, Critical Care and Pain Medicine, Massachusetts General Hospital, Harvard Medical School, Charlestown, MA 02129 USA; 2 Shriners Hospitals for Children, Boston, MA 02114 USA; 3 Department of Anesthesiology, Kyorin University School of Medicine, Tokyo, Japan; 4 Division of Gene Regulation and Signal Transduction, Research Center for Genomic Medicine, Saitama Medical University, Saitama, Japan; 5 Department of Geriatrics and Vascular Medicine, Tokyo Medical and Dental University Graduate School, Tokyo, Japan; 6 Department of Surgery, Massachusetts General Hospital, Harvard Medical School, Boston, MA 02114 USA; 7 Tokyo Metropolitan Institute of Gerontology, Tokyo, Japan

## Abstract

Metabolic derangements are a clinically significant complication of major trauma (e.g., burn injury) and include various aspects of metabolism, such as insulin resistance, muscle wasting, mitochondrial dysfunction and hyperlactatemia. Nonetheless, the molecular pathogenesis and the relation between these diverse metabolic alterations are poorly understood. We have previously shown that burn increases farnesyltransferase (FTase) expression and protein farnesylation and that FTase inhibitor (FTI) prevents burn-induced hyperlactatemia, insulin resistance, and increased proteolysis in mouse skeletal muscle. In this study, we found that burn injury activated mTORC1 and hypoxia-inducible factor (HIF)-1α, which paralleled dysfunction, morphological alterations (i.e., enlargement, partial loss of cristae structure) and impairment of respiratory supercomplex assembly of the mitochondria, and ER stress. FTI reversed or ameliorated all of these alterations in burned mice. These findings indicate that these burn-induced changes, which encompass various aspects of metabolism, may be linked to one another and require protein farnesylation. Our results provide evidence of involvement of the mTORC1-HIF-1α pathway in burn-induced metabolic derangements. Our study identifies protein farnesylation as a potential hub of the signaling network affecting multiple aspects of metabolic alterations after burn injury and as a novel potential molecular target to improve the clinical outcome of severely burned patients.

## Introduction

Metabolic derangements in skeletal muscle are a clinically important complication of major trauma, such as burn injury, and affect the clinical trajectory of patients with major trauma. These metabolic alterations include various aspects of metabolism, such as hyperlactatemia, insulin resistance, muscle wasting, and mitochondrial dysfunction^1–6^. The molecular mechanisms that underlie these alterations are incompletely understood. Moreover, limited knowledge is available about the relation between these metabolic derangements.

Hyperlactatemia is a hallmark of critically ill patients, including those with severe burn injury^6–8^. Hyperlactatemia is an independent risk factor for the mortality of burn patients^7^. Over and above hypoperfusion and subsequent tissue hypoxia, a metabolic shift from mitochondrial oxidative phosphorylation to ATP synthesis by glycolysis in the presence of sufficient oxygen availability has been proposed to contribute to hyperlactatemia in critical illnesses, such as sepsis and burn injury^9–13^. The predominance of glycolysis over mitochondrial oxidative phosphorylation under normoxia was discovered in cancer cells by Dr. Otto H. Warburg in the 1920s, referred to as the Warburg effect (also known as aerobic glycolysis). Hypoxia-inducible factor (HIF)-1α is a master transcription factor that orchestrates the Warburg effect by upregulating the transcription of glycolytic genes, such as glucose transporter-1 (Glut1) and pyruvate dehydrogenase kinase-1 (PDK1), and thereby increases glycolysis, leading to increased lactate production^14^.

Essentially the same metabolic shift has been termed “cytopathic hypoxia” in critical illness, although the role of HIF-1α in cytopathic hypoxia has not been studied^9, 10^. Several recently accumulating lines of evidence indicate that the Warburg effect is not specific to cancer cells, but can be induced by inflammation in non-transformed cells^15, 16^. Increased lactate production plays an important role in the hyperlactatemia associated with critical illness. Skeletal muscle is the major site of lactate production in our body. Burn injury increases lactate production by skeletal muscle^17, 18^. These finding raise the possibility that activation of the HIF-1α pathway may play a role in the metabolic shift (namely cytopathic hypoxia) in skeletal muscle in critical illness and major trauma. However, this possibility has not yet been studied in critical illness, including burn injury. In fact, it is not known whether HIF-1α is induced by major trauma (e.g., burn injury).

Activation of mammalian target of rapamycin complex 1 (mTORC1) induces HIF-1α expression in cultured cells^19–22^. Moreover, mTORC1 activation plays a critical role in obesity-induced insulin resistance^23^. On the other hand, the impact of major trauma (e.g., burn injury) on mTORC1 has not been extensively studied, although we have previously shown that phosphorylation (activation) of mTOR and p70 S6 kinase (p70S6K) were increased at 3 days after burn injury in rats^24^. In contrast to obesity-induced insulin resistance and burn injury, previous studies have shown that sepsis decreases mTORC1 activity in skeletal muscle of rodents^25, 26^. Whereas both burn injury and sepsis lead to insulin resistance, muscle wasting, and hyperlactatemia, the role of mTORC1 in metabolic alterations may differ between burn injury and sepsis.

Furthermore, the relation between hyperlactatemia and insulin resistance is not fully understood. Insulin resistance can cause hyperlactatemia in mice. Muscle-specific knockout of insulin receptor substrate (IRS)-1 and IRS-2 caused insulin resistance and hyperlactatemia in mice, although neither hyperglycemia nor glucose intolerance was induced^27^. Conversely, hyperlactatemia can induce insulin resistance in skeletal muscle in rodents^28, 29^. Nonetheless, the molecular mechanisms by which insulin resistance induces hyperlactatemia and vice versa remain largely unknown.

Cachexia is a complex metabolic syndrome associated with underlying illness and characterized by loss of skeletal muscle mass and increased proteolysis^30, 31^. Cachexia has been considered one of the primary causes of mortality of cancer patients. Similarly, cachectic changes, such as muscle wasting, increased protein breakdown, and body weight loss, have been recently proposed to constitute a serious disease state that dictates the clinical outcome of critically ill patients, including those with burn injury^3, 32, 33^ and sepsis^34^. Inflammation, insulin resistance, and mitochondrial dysfunction are associated with cachexia^3, 33, 35, 36^. However, limited knowledge is available about the molecular pathogenesis of cachexia. Little is known about the mechanisms by which cachectic changes, such as muscle wasting and increased protein breakdown, are induced by major trauma (e.g., burn injury).

Protein farnesylation is a posttranslational modification of a cysteine residue that is catalyzed by farnesyltransferase (FTase). FTase catalyzes the covalent attachment of the farnesyl group to cysteine thiol in the CAAX motif located in the carboxyl terminus, where C represents cysteine, A is any aliphatic acid, and X is any amino acid in the carboxyl terminus. In an attempt to clarify the molecular mechanisms by which burn injury induces metabolic derangements and insulin resistance in skeletal muscle, we have previously shown the important role of protein farnesylation^17^. Burn injury increases FTase expression and the abundance of farnesylated proteins in mouse skeletal muscle. Of note, burn-induced increased FTase expression parallels the development of insulin resistance and hyperlactatemia^17, 37^. The maximum effects of burn injury on insulin resistance, hyperlactatemia, and FTase expression were observed at 3 days after burn injury compared with sham-burn injury, while significant alterations in plasma lactate, insulin signaling, and FTase expression were not observed at 1 day after burn injury compared with naïve control mice^17, 37^. A competitive inhibitor for FTase, FTI-277, prevents burn-induced metabolic alterations, including insulin resistance, increases in lactate production, and protein breakdown in mouse skeletal muscle. To further study the molecular mechanisms that underlie the protective effects of FTI-277 and the relation between metabolic alterations and insulin resistance, we examined the effects of burn and FTI-277 on the HIF-1α pathway and mitochondrial function and morphology.

## Results

### Burn injury induced HIF-1α expression in mouse skeletal muscle

**Figure 1:**
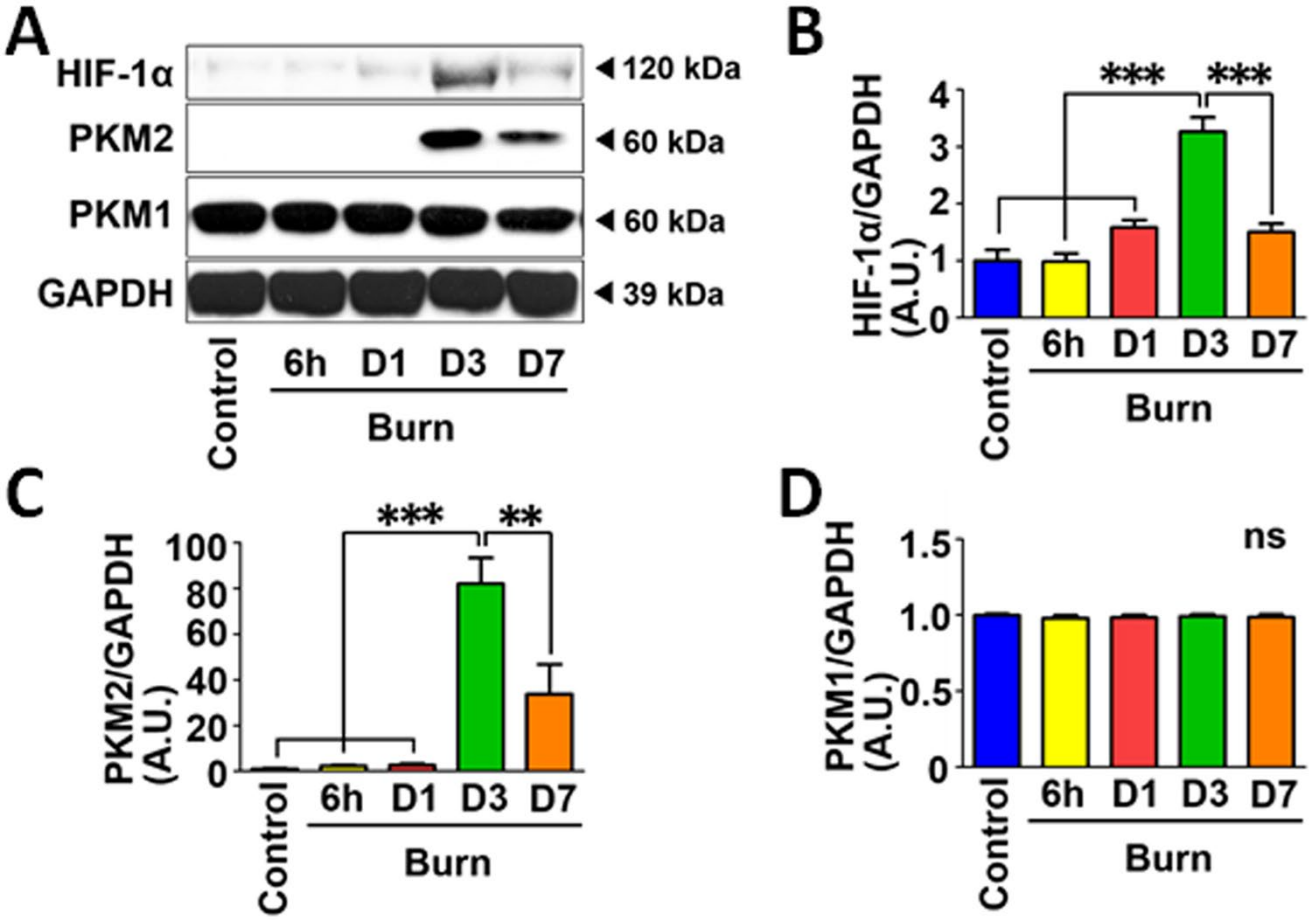
HIF-1α and PKM2 expression was induced in skeletal muscle at 3 days after burn injury. Protein expression of HIF-1α, PKM2, and PKM1 was examined in skeletal muscle of naïve (Control) mice and burned mice at 6 h, 1, 3 and 7 days after burn. HIF-1α and PKM2 protein expression was significantly increased at 3 days after burn (**A**–**C**). Burn injury did not alter protein expression of PKM1 (**D**). **P < 0.01, ***P < 0.001. ns: not significant. n = 4 mice per group.

In our previous study, burn injury induced delayed hyperlactatemia at 3 days after burn injury compared with naïve (control) mice, whereas the plasma lactate level was not significantly increased at 6 h and 1 day post-burn^17^. Consistently, HIF-1α protein expression was significantly increased in mouse skeletal muscle at 3 days after burn compared with naïve mice, although HIF-1α expression was not increased at 6 h post-burn (Fig. 1A and B). At 1 and 7 days post-burn injury, there were trends toward increased HIF-1α expression relative to naïve mice, but there were no statistically significant differences. In parallel with HIF-1α induction, pyruvate kinase muscle isozyme 2 (PKM2) protein expression was markedly induced at 3 days after burn injury (Fig. 1A and C). PKM2 is a downstream gene of HIF-1α and plays an important role in aerobic glycolysis and increased lactate production^38^. The burn-induced expression of PKM2 is consistent with activation of HIF-1α after burn injury. In contrast, PKM2 expression was undetectable in skeletal muscle of naïve mice and at 6 h and 1 day after burn. These results are consistent with previous studies which indicate that PKM1, but not PKM2, is expressed in skeletal muscle in mice under a normal condition^39^. PKM2 expression seemed to be increased at 7 days post-burn injury, although the apparent increase in PKM2 expression at 7 days post-burn did not achieve statistical significance in comparison with naïve mice (Fig. 1A and C). On the other hand, PKM1 expression was not altered after burn injury (Fig. 1A and D).

### FTI-277 prevented burn-induced activation of the HIF-1α pathway

**Figure 2:**
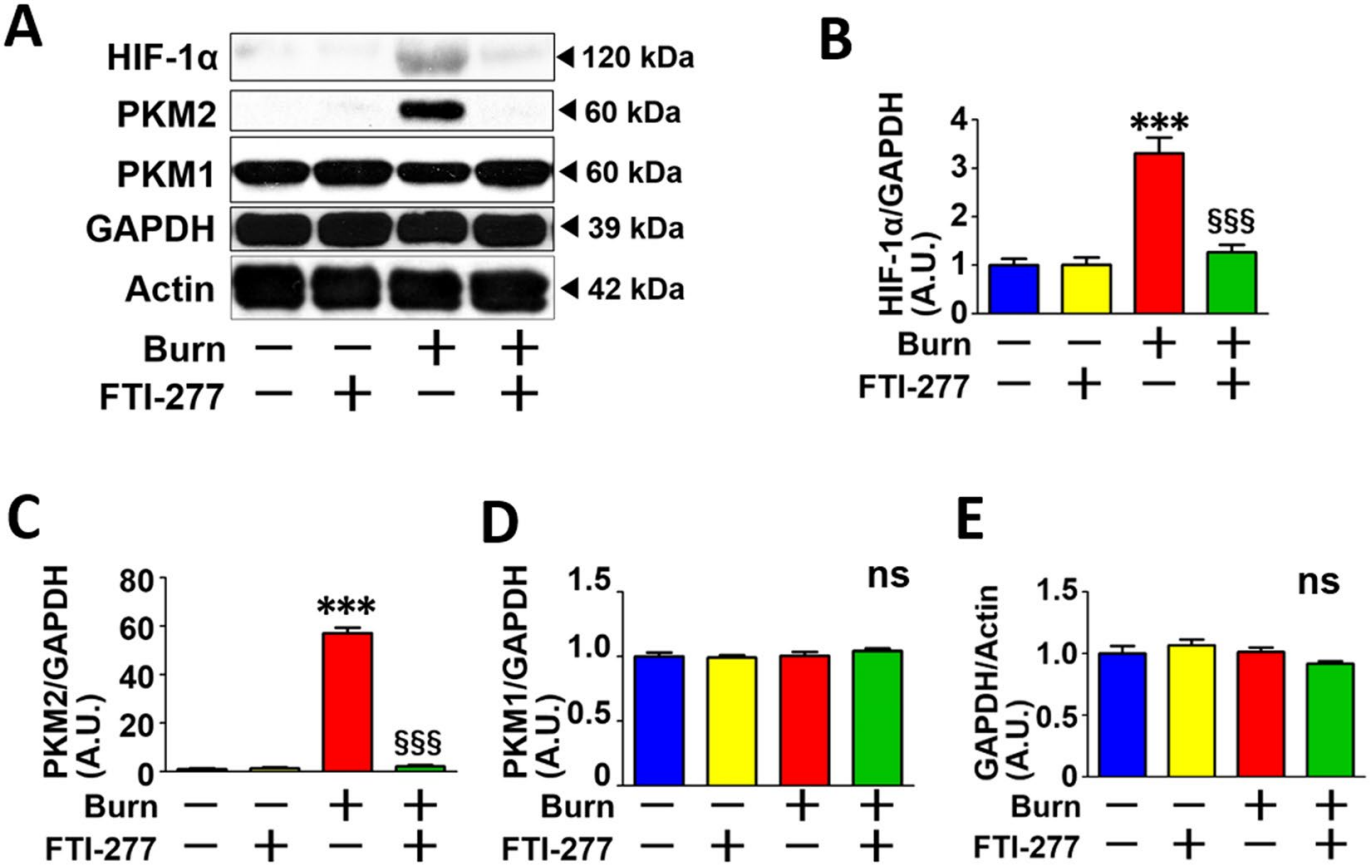
FTI-277 treatment reversed burn-induced activation of the HIF-1α pathway in skeletal muscle. Burn injury significantly increased HIF-1α and PKM2 protein expression in mouse skeletal muscle at 3 days post-burn. FTI-277 treatment reversed burn-induced increased HIF-1α and PKM2 expression (**A**–**C**). PKM1 and GAPDH protein expression was not altered by burn injury or FTI-277 treatment (**A**,**D** and **E**). ***P < 0.001 vs. sham-burn groups, ^§§§^P < 0.001 vs. vehicle-treated burn group. ns: not significant. n = 6 mice per group.

**Figure 3:**
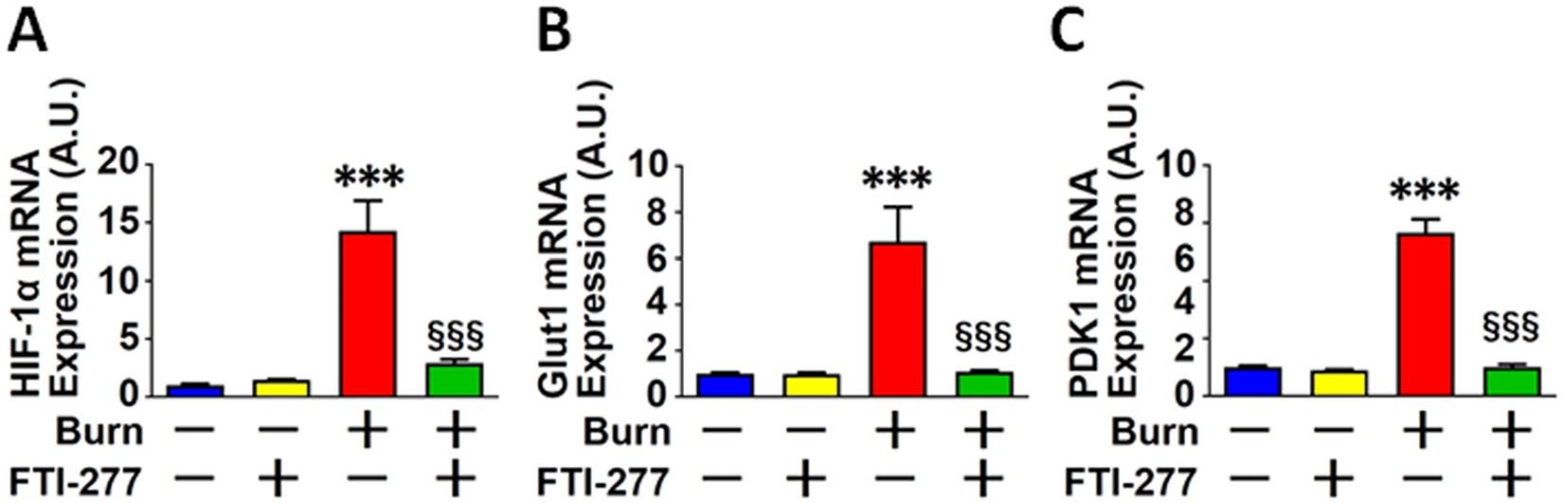
FTI-277 treatment reversed burn-induced induction of HIF-1α, Glut1, and PDK1 genes expression in skeletal muscle. mRNA levels of HIF-1α, Glut1, and PDK1 in skeletal muscle were significantly increased at 3 days after burn. FTI-277 treatment prevented these alterations (**A**–**C**). ***P < 0.001 vs. sham-burn groups, ^§§§^P < 0.001 vs. vehicle-treated burn group. n = 6 mice per group.

FTI-277 treatment reversed burn-induced expression of HIF-1α and PKM2 at 3 days after burn injury in mouse skeletal muscle compared with vehicle alone (Fig. 2A, B and C). These findings are consistent with our previous study which showed that FTI-277 prevented burn-induced hyperlactatemia and increased lactate production by skeletal muscle in mice at 3 days post-burn injury^17^. On the other hand, neither burn injury nor FTI-277 altered the protein expression of PKM1 or GAPDH (Fig. 2A, D and E). Burn injury increased mRNA expression of HIF-1α and genes involved in basal (insulin-independent) glucose uptake and glycolysis, Glut1 and PDK1^40, 41^, which are the downstream targets of HIF-1α (Fig. 3A–C). FTI-277 inhibited burn-induced increases in mRNA levels of HIF-1α, Glut1, and PDK1 (Fig. 3A–C). These results indicate that FTI-277 prevented burn-induced activation of the HIF-1α pathway.

### Burn injury increased mTORC1 activity and FTI-277 reversed it

**Figure 4:**
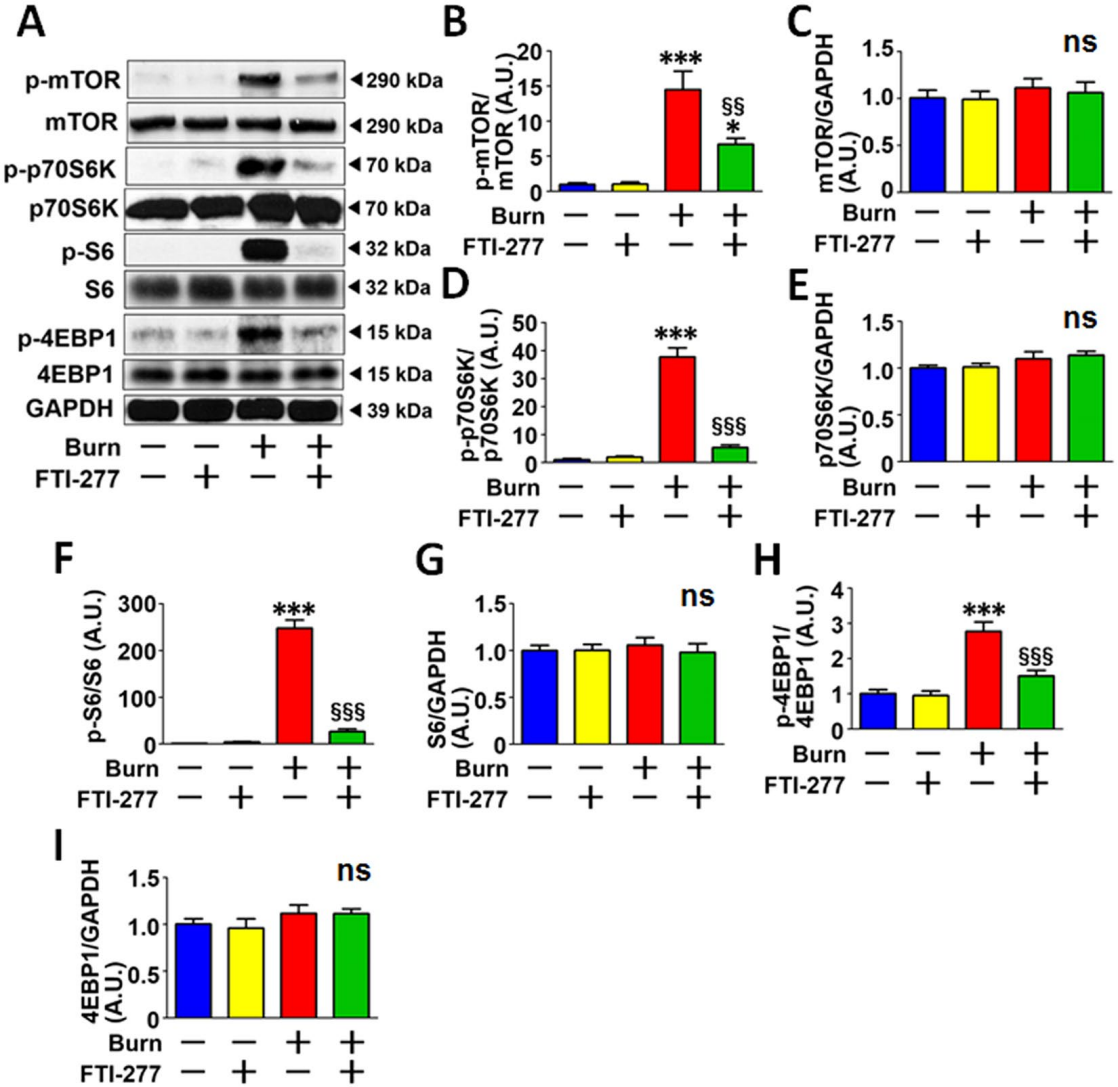
FTI-277 treatment inhibited burn-induced mTORC1 activation in skeletal muscle. Phosphorylation of mTOR, p70S6K, S6, and 4EBP1, which were normalized to total mTOR, p70S6K, S6, and 4EBP1, respectively, was significantly increased in skeletal muscle at 3 days after burn injury compared with sham-burn. FTI-277 significantly inhibited burn-induced increased phosphorylation of these molecules (**A**,**B**,**D**,**F**,**H**). Protein expression of total mTOR, p70S6K, S6, and 4EBP1 was not altered by burn or FTI-277 (**A**,**C**,**E**,**G**,**I**). *P < 0.05, ***P < 0.001 vs. sham-burn groups, ^§§^P < 0.01, ^§§§^P < 0.001 vs. vehicle-treated burn group. ns: not significant. n = 6 mice per group.

Previous studies have shown that mTORC1 activation induces HIF-1α in the absence of hypoxia in cultured cells^19–21^. We, therefore, examined the effects of burn injury and FTI-277 on mTORC1 activity. Burn injury markedly increased phosphorylation of mTOR, p70S6K, S6, and 4EBP1 in mouse skeletal muscle (Fig. 4). On the other hand, protein expression of mTOR, p70S6K, S6, and 4EBP1 was not altered by burn. These results indicate that burn injury increased mTORC1 activity.

FTI-277 inhibited burn-induced mTORC1 activation compared with vehicle alone, as indicated by the inhibition of increases in phosphorylation of mTOR, p70S6K, S6, and 4EBP1 in burned mice (Fig. 4). FTI-277 did not alter phosphorylation of these proteins in sham-burned mice or protein expression of mTOR, p70S6K, S6, or 4EBP1 regardless of burn or sham-burn injury.

We wanted to ask whether increased protein phosphorylation after burn is specific to components of mTORC1 or a generally observed phenomenon in most proteins. In the attempt to address this question, we evaluated phosphorylation of mitogen-activated protein kinases (MAPKs). Burn injury increased phosphorylation of extracellular signal-regulated kinase (ERK), decreased phosphorylation of p38 MAPK, and did not alter phosphorylation of c-Jun N-terminal protein kinase (JNK) compared with sham-burn (Supplementary Figure 1). ERK expression was increased by burn injury. Protein expression of p38 MAPK and JNK was not altered by burn injury. These results indicate that the impact of burn injury on phosphorylation status varies dependent on the protein of interest.

### FTI-277 ameliorated burn-induced mitochondrial dysfunction

**Figure 5:**
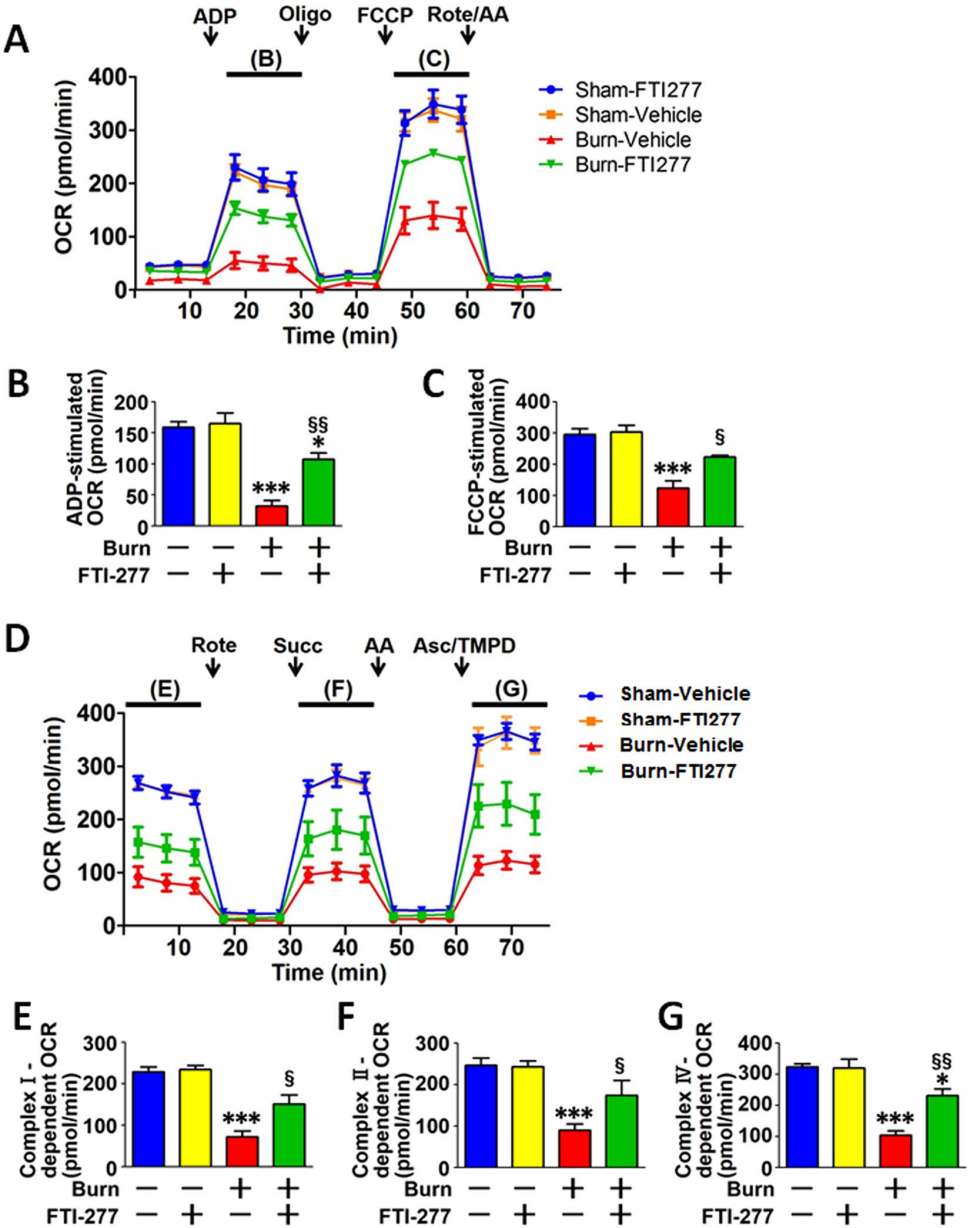
FTI-277 ameliorated burn-induced decreases in mitochondrial oxygen consumption rate (OCR) in skeletal muscle. ADP- and FCCP-stimulated OCRs were significantly decreased by burn injury at 3 days post-burn, both of which were ameliorated by FTI-277 treatment (**A**–**C**). Similarly, complex I-, complex II-, and complex IV-dependent OCRs were significantly decreased at 3 days after burn injury, all of which were ameliorated by FTI-277 (**D**–**G**). The time points, in which ADP- (**B**), FCCP- (**C**), complex I- (**E**), complex II- (**F**), and complex IV- (**G**) dependent OCRs were evaluated, are indicated in A and C. ADP: adenosine diphosphate, Oligo: oligomycin, FCCP: carbonyl cyanide 4-(trifluoromethoxy) phenylhydrazone, Rote: rotenone, AA: antimycin A, Succ: succinate, Asc: ascorbate, TMPD: N, N, N9, N9-Tetramethylp-phenylenediamine. *P < 0.05, ***P < 0.001 vs. sham-burn groups, ^§^P < 0.05, ^§§^P < 0.01 vs. vehicle-treated burn group. n = 4 mice per group.

Burn injury causes mitochondrial dysfunction^2–4^. Next, we studied the effects of FTI on mitochondrial dysfunction in mouse skeletal muscle. Burn injury suppressed ADP-stimulated oxygen consumption rate (OCR) and the maximum OCR capacity, the latter of which was evaluated by measuring OCR stimulated by FCCP, a mitochondrial oxidative phosphorylation uncoupler (Fig. 5A, B and C). FTI-277 significantly ameliorated burn-induced decreases in ADP-stimulated and FCCP-stimulated OCRs (Fig. 5A, B and C).

Next, we evaluated complex I-, complex II-, and complex IV-dependent electron transfer capacity by measuring rotenone-inhibitable, succinate-stimulated, and ascorbate-plus-TMPD-stimulated OCRs, respectively. Burn injury markedly suppressed complex I-, complex II-, and complex IV-dependent OCRs in mouse skeletal muscle compared with sham-burn. FTI-277 significantly ameliorated the burn-induced decreases in the OCRs (Fig. 5D, E, F and G). These data indicate that burn injury induced a general decrease in OCRs of various components of the electron transport chain to a comparable degree. FTI-277 did not alter mitochondrial function in sham-burned mice. Together, these results indicate that FTI-277 mitigated burn-induced suppression of mitochondrial oxidative phosphorylation.

### FTI-277 prevented burn-induced morphological alterations of the mitochondria

**Figure 6:**
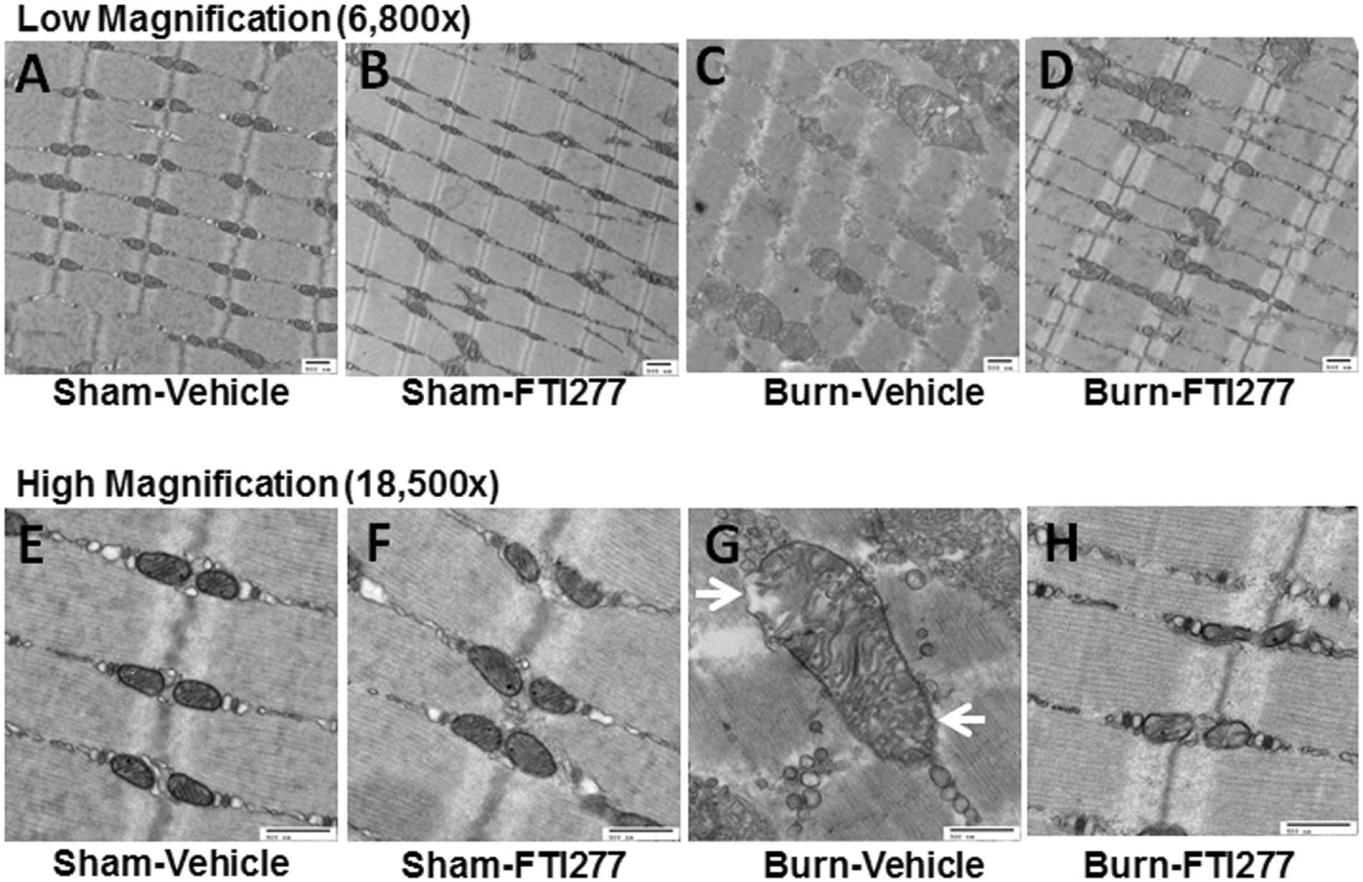
FTI-277 prevented burn-induced mitochondrial morphological changes in skeletal muscle. Enlarged mitochondria were observed in skeletal muscle at 3 days after burn injury, although enlarged mitochondria were not found in sham-burned mice. In addition, burn injury resulted in loss of cristae structure in some area of the mitochondria compared with sham-burn. FTI-277 prevented these morphological alterations induced by burn injury. On the other hand, FTI-277 did not affect the mitochondrial morphology in sham-burned mice (**I**–**K**). Arrows indicate the area with loss of cristae structure (**G**). (**A**–**D**): low magnification (6,800x), (**E**–**H**): high magnification (18,500x). scale bar: 500 nm. (**A**,**E**): sham-vehicle. (**B**,**F**): sham-FTI. (**C**,**G**): burn-vehicle. (**D**,**H**): burn-FTI.

Next, we studied the effects of burn injury and FTI-277 on the morphology of the mitochondria in mouse skeletal muscle at 3 days after burn injury. Transmission electron microscopy revealed that burn injury resulted in morphological alterations of the mitochondria compared with sham-burn (Fig. 6C and G). Specifically, enlarged mitochondria (such as those with area >0.8 μm^2^) were observed in skeletal muscle of burned mice, although they were not found in sham-burned mice (Average size of the mitochondria [μm^2^]: Sham + Vehicle: 0.11; Sham + FTI: 0.15; Burn + Vehicle: 0.77; Burn + FTI: 0.19). The numbers of the mitochondria were 27, 26, 13, and 23 per 100 μm^2^ myofiber area in vehicle- and FTI-277-treated sham-burned mice and vehicle- and FTI-277-treated burned mice, respectively. The mitochondrial density was greater in vehicle-treated burned mice relative to the other groups (Mitochondrial density [%]: Sham + Vehicle: 3.7; Sham + FTI: 5.0; Burn + Vehicle: 12.1, Burn + FTI: 5.5). In addition, burn injury induced loss of cristae structure in some area of the mitochondria compared with sham-burn (Average length of cristae/mitochondrial area [μm/μm^2^]: Sham + Vehicle: 13.3; Sham + FTI: 12.7; Burn + Vehicle: 6.8; Burn + FTI: 11.6). FTI-277 inhibited these morphological alterations induced by burn injury. On the other hand, FTI-277 did not affect the mitochondrial morphology in sham-burned mice.

**Figure 7:**
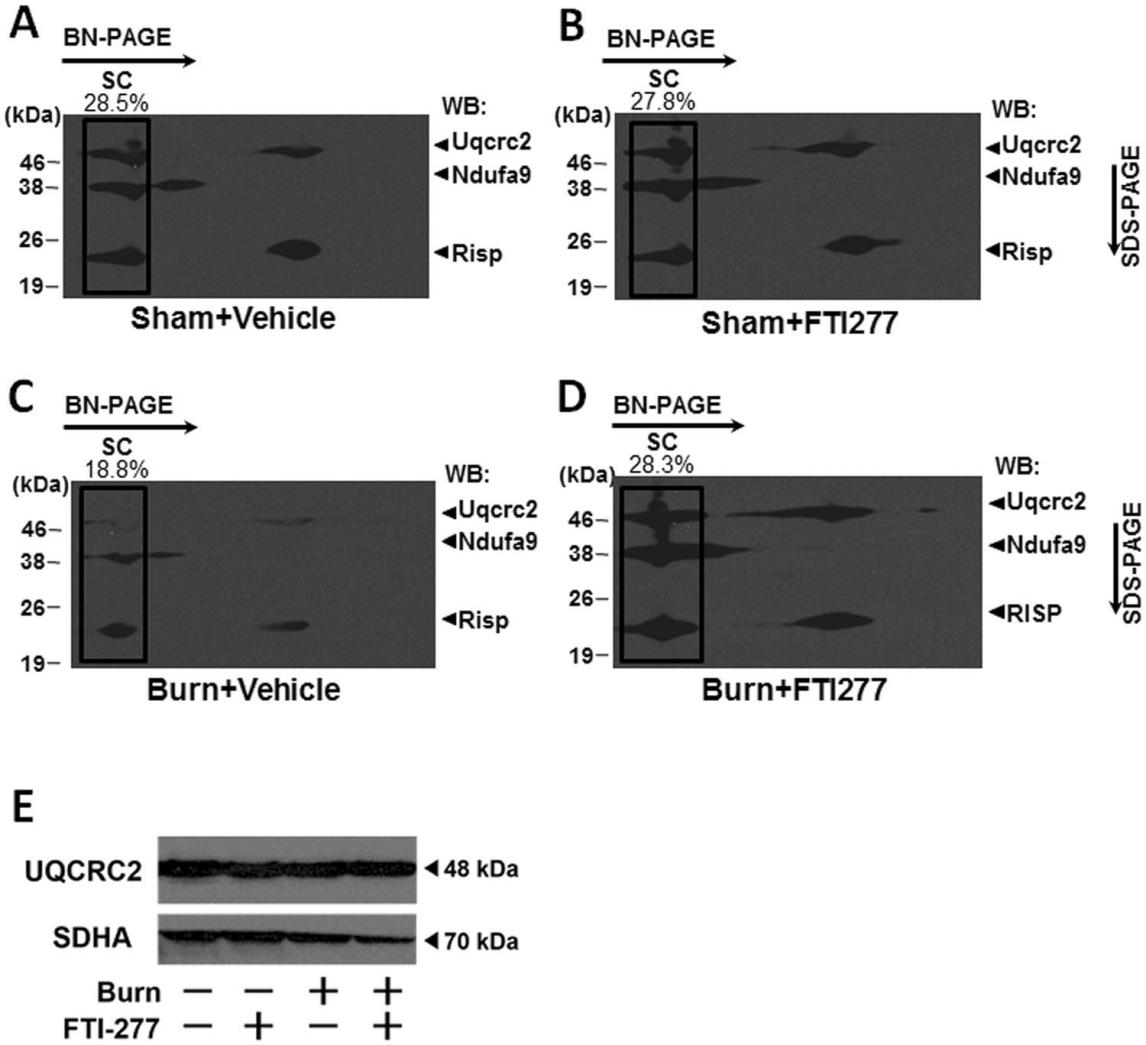
FTI-277 prevented burn-induced impaired mitochondrial respiratory supercomplex assembly in skeletal muscle. At 3 days after burn or sham-burn, respiratory supercomplexes in the mitochondria in skeletal muscle were separated by blue native (BN) PAGE followed by second-dimensional SDS-PAGE and visualized using antibodies for NDFUA9 (a component of complex I), and UQCRC2 and RISP (components of complex III). The percentage of the complex I-containing largest supercomplex (SC) signal intensity was decreased in vehicle-treated burned mice compared with sham-burned mice. FTI-277 treatment inhibited the burn-induced alteration in the supercomplexes. On the other hand, total protein abundance of UQCRC2 was not altered by burn injury or FTI-277 (**E**). SDHA (a component of complex II) was used as a control. WB: Western blotting.

In the mitochondria, complex I, complex III, and complex IV form respiratory supercomplexes, which are important for efficient electron transport and oxidative phosphorylation^42, 43^. Conversely, disruption of respiratory supercomplexes leads to a decrease in oxidative phosphorylation capacity^42, 43^. Moreover, respiratory supercomplexes exist in the cristae of the mitochondria, and loss of cristae structure has been proposed to be linked to decreased formation of respiratory supercomplexes^44^. We, therefore, studied the effects of burn injury and FTI-277 on respiratory supercomplexes in the mitochondria in mouse skeletal muscle by two-dimensional blue native PAGE (2D-BN-PAGE). As expected, several respiratory supercomplexes were detected in mouse skeletal muscle, including those consisting of: (1) complex I, complex III, and complex IV; and (2) complex III and complex IV^45^. The largest supercomplex is the only supercomplex that consists of complex I, complex III, and complex IV. When treated with vehicle alone, burn injury decreased the percentage of the signal intensity of the complex I-containing largest supercomplex relative to all the signals in the blot compared with sham-burned mice (Fig. 7A–D). FTI-277 restored the formation of respiratory supercomplexes in burned mice (Fig. 7A–D). All the respiratory supercomplexes included complex III. Total protein abundance of UQCRC2, a component of complex III, was not altered by burn injury or FTI-277 (Fig. 7E). These data indicate that respiratory supercomplex formation was impaired by burn injury and restored by FTI-277. On the other hand, FTI-277 did not alter respiratory supercomplexes in sham-burned mice.

### FTI-277 inhibited burn-induced ER stress in mouse skeletal muscle

**Figure 8:**
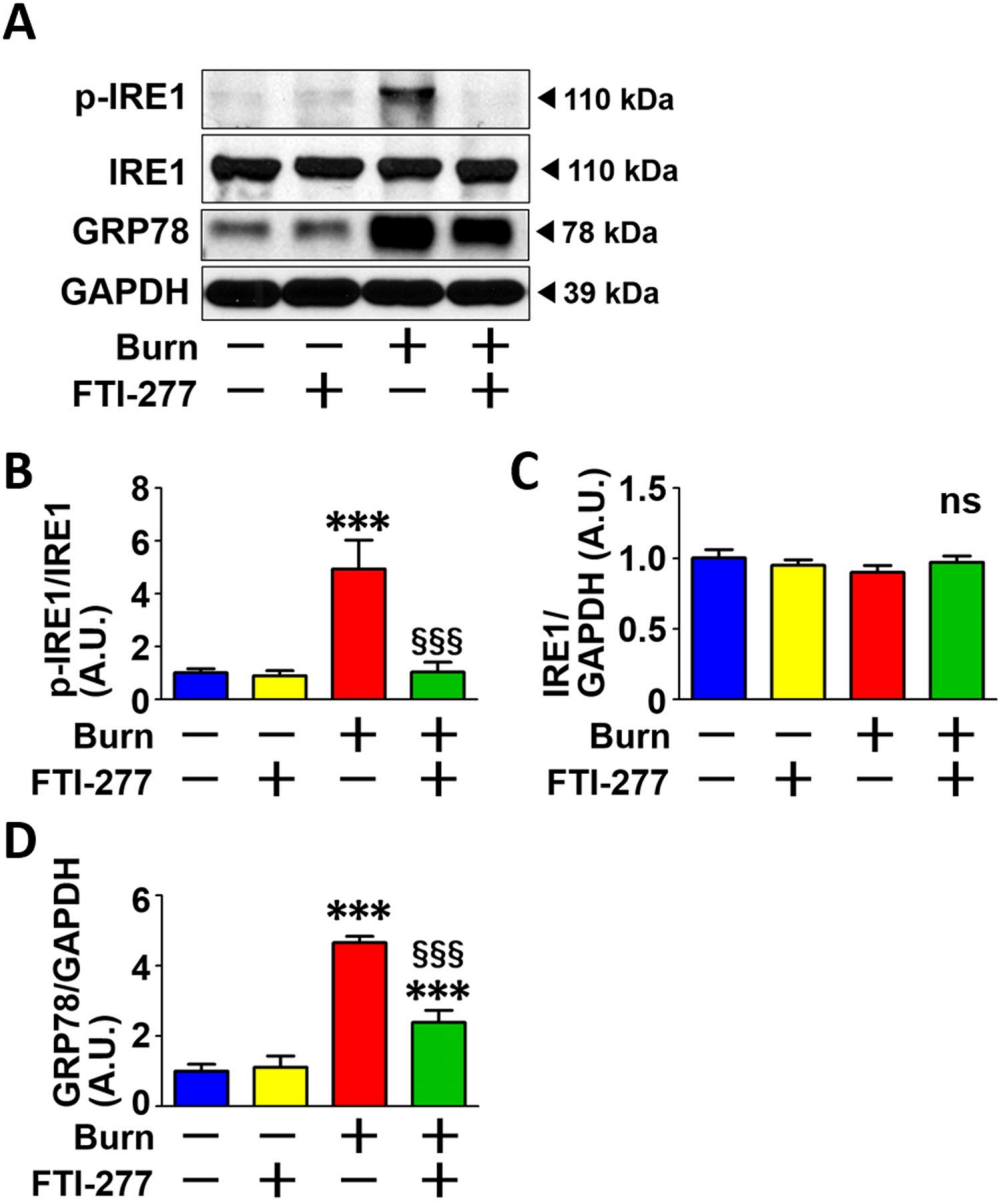
FTI-277 inhibited burn-induced ER stress in skeletal muscle. Burn injury significantly increased phosphorylation of IRE1 (**A**,**B**) and Grp78 protein expression (**A**,**D**) compared with sham-burn. FTI-277 mitigated burn-induced increases in phosphorylation of IRE1 and protein expression of Grp78. IRE1 protein expression was not altered by burn injury or FTI-277. ***P < 0.001 vs. sham-burn groups, ^§§§^P < 0.001 vs. vehicle-treated burn group. n = 6 mice per group.

The mitochondria and endoplasmic reticulum (ER) physically and functionally interact with each other within cells^46^. Consistently, dysfunction and morphological alteration of the mitochondria are often associated with ER stress^47^. To assess ER stress, we evaluated phosphorylation of inositol-requiring enzyme 1 (IRE1), a sensor of ER stress^48^, and expression of glucose-regulated protein (GRP78) (also known as BiP), a master regulator of the unfolded protein response^49^. Burn markedly increased phosphorylation of IRE1 and Grp78 protein expression compared with sham-burn. The burn-induced ER stress was consistent with previous studies which indicate that burn injury causes ER stress in the liver^50, 51^. FTI-277 inhibited burn-induced increases in phosphorylation of IRE1 and protein expression of Grp78 (Fig. 8A, B and D). IRE1 protein expression was not altered by burn injury or FTI-277 (Fig. 8A and C).

## Discussion

Here, we show in mice that burn injury induced activation of the HIF-1α pathway and mTORC1, ER stress, and dysfunction and morphological alterations of the mitochondria in skeletal muscle compared with sham-burn. HIF-1α protein expression was induced at 3 days after burn injury, the time point at which the maximum effects of burn injury on insulin resistance and plasma lactate levels were observed in our previous studies using the same mouse model of burn injury^17, 36^.

FTI-277 blocked the activation of the HIF-1α pathway, almost completely inhibited mTORC1 activation, and mitigated ER stress in skeletal muscle of burned mice. Simultaneously, FTI-277 ameliorated mitochondrial dysfunction, morphological alterations, and impaired formation of respiratory supercomplexes in the mitochondria in burned mice. In our previous study, burn injury increased the amount of farnesylated proteins in mouse skeletal muscle, but this was reversed by FTI-277^17^. In parallel, FTI-277 prevented burn-induced insulin resistance (i.e., impaired insulin signaling, suppressed insulin-stimulated glucose uptake), increases in lactate production and proteolysis in skeletal muscle, and hyperlactatemia in mice^17^. Together, these results indicate that protein farnesylation plays an important role in the various effects of burn, namely, metabolic alterations, mitochondrial dysfunction, and activation of multiple signaling pathways (i.e., the HIF-1α pathway, mTORC1, and ER stress) in mouse skeletal muscle.

Insulin resistance in skeletal muscle can cause increased lactate production by skeletal muscle and hyperlactatemia^27^. Conversely, hyperlactatemia can induce insulin resistance in skeletal muscle^28, 29^. Insulin resistance contributes to increased proteolysis and muscle wasting^52, 53^. Thus, these metabolic alterations are unlikely to represent separate events that occur coincidentally at the same time point after burn injury. Rather, they appear to be intrinsically related to each other via some yet to be determined mechanism(s). However, the precise relation between the various aspects of burn-induced muscle metabolic derangements, such as insulin resistance, proteolysis, and lactate production, remains to be clarified. Based on our previous study which demonstrated that FTI-277 reverses all of these metabolic alterations in burned mice^17^, one can speculate that a common mechanism, in which protein farnesylation plays an essential role, is involved in the metabolic alterations after burn injury.

HIF-1α is a master transcription factor that orchestrates the Warburg effect (also known as aerobic glycolysis)^14^. The Warburg effect was originally described as the metabolic reprogramming of cancer cells and is characterized by increased glycolysis and lactate production under normoxic conditions. Recent studies have shown that the Warburg effect is not specific to cancer cells, but can also be induced by inflammation^15, 16^. Essentially, the “aerobic glycolysis of cancer” and the “cytopathic hypoxia of critical illness” are different terms for the same metabolic shift^9, 10^. Whether HIF-1α induction is associated with cytopathic hypoxia in critical illness, including burn injury, has not been investigated. Our study provides the first evidence indicating a role of activation of the HIF-1α pathway and the Warburg-like effect (cytopathic hypoxia) in burn-induced metabolic derangements.

In this study, burn injury induced mitochondrial dysfunction, activation of the HIF-1α pathway, activation of mTORC1, and ER stress in mouse skeletal muscle, all of which were inhibited by FTI-277. Interaction and crosstalk have been reported between these changes, although the specific interactions have not been studied in burn injury or major trauma. For example, suppression of mitochondrial oxidative phosphorylation leads the HIF-1α pathway activation to increase glycolytic ATP synthesis. Conversely, activation of the HIF-1α pathway decreases mitochondrial function^54^. mTORC1 activation can induce HIF-1α^19–22^. On the other hand, HIF-1α-mediated metabolic reprograming has been shown to activate mTORC1^55^ although previous studies have also shown that HIF-1α inhibits mTORC1 activity in a different cellular context^56^. mTORC1 activation causes or exacerbates ER stress^57^. ER stress increases the activity of the HIF-1α pathway^58, 59^. ER stress and mitochondrial dysfunction are closely related to each other^47^. A simple linear model, in which upstream and downstream events are clearly differentiated, may be inadequate to decipher the crosstalk and reciprocal up- or down-regulation that occurs between mTORC1, the HIF-1α pathway, ER, and the mitochondria. Rather, it is possible that these changes might enhance one another through positive feedback and feed-forward mechanisms, reaching a critical point at 3 days post-burn injury. It is reasonable to speculate, therefore, that a vicious, self-reinforcing cycle, which is composed of the activation of HIF-1α and mTORC1, ER stress response, and mitochondrial dysfunction, may underlie the evolvement of various aspects of the burn-induced metabolic derangements. Recently, the close link between inflammation and metabolic alterations has been increasingly recognized as a major event in the development of many disease conditions. Persistent inflammation and metabolic dysfunction enhance each other, forming a metabolic inflammatory complex, which, in turn, contributes to the development of many human diseases, including sepsis and major trauma^34^. Collectively, our data suggest that protein farnesylation may be a driver of the vicious cycle that causes metabolic inflammatory complex in burn injury.

mTORC1 activation plays a pivotal role in obesity-induced insulin resistance in mice^60^. We have previously shown that burn injury increased phosphorylation of mTOR and p70S6K in skeletal muscle at 3 days after burn in rats^23^, consistent with the findings in the present study in mice. In contrast, previous studies have revealed that mTORC1 activity is decreased in skeletal muscle of septic rodents^25, 26^, which, in turn, contributes to sepsis-induced muscle wasting by decreasing protein synthesis. It is important to note that mTORC1 activation as well as decreased mTORC1 activity may promote muscle wasting. A previous study has shown that mTORC1 activation mediates denervation-induced muscle atrophy by inhibiting the Akt signaling pathway^61^. Of note, denervation-induced decrease in Akt activity is reminiscent of decreased insulin-stimulated Akt activation in skeletal muscle of burned mice^17, 37^, which can be reversed by FTI-277^17^. Together, it is conceivable that the role of mTORC1 in muscle wasting may differ between sepsis and burn injury, although dysregulated mTORC1 activity is associated with muscle wasting in these disease conditions.

Loss of cristae structure is associated with mitochondrial dysfunction^62, 63^. Respiratory supercomplexes are localized in the cristae of the mitochondria^43^. Loss of cristae structure leads to disruption of respiratory supercomplexes, contributing to mitochondrial dysfunction^44^. Consistently, the formation of respiratory supercomplexes was impaired in parallel with the partial loss of cristae structure and decreased oxidative phosphorylation capacity of the mitochondria after burn injury. FTI-277 mitigated these alterations in the mitochondria in burned mice.

Although immune cells, such as neutrophils, infiltrate into skeletal muscle tissue after burn injury, immunoblot analysis with antibody for neutrophil elastase revealed that the level of neutrophils in skeletal muscle of burned mice was less than 5% of the levels found in spleen of naïve mice (Supplementary Figure 2). These results suggest that most of the proteins in the muscle homogenates, which were used for immunoblotting in this study, were derived from skeletal muscle cells rather than infiltrated immune cells, and that the burn-induced changes in the proteins tested are mainly attributable to the alterations in skeletal muscle cells.

**Figure 9:**
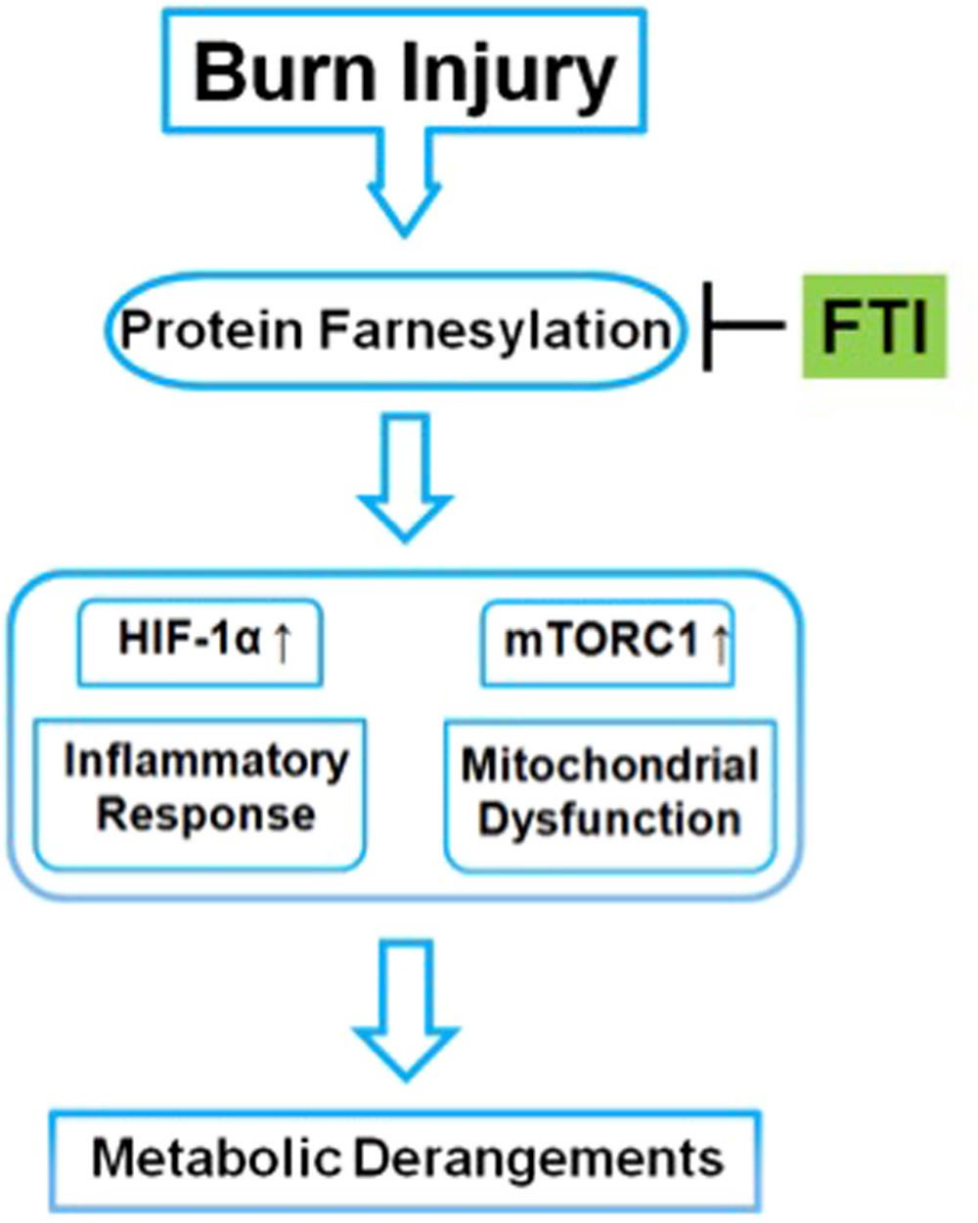
Schematic presentation of the proposed model: Role of protein farnesylation in burn-induced metabolic derangements. Our data indicate that protein farnesylation causes and/or exacerbates activation of the HIF-1α pathway and mTORC1, mitochondrial dysfunction, and ER stress, all of which contribute in concert to burn-induced muscle metabolic derangements, including insulin resistance and increases in lactate production and protein breakdown. Farnesyltransferase (FTase) inhibitor (FTI) prevents burn-induced metabolic derangements by inhibiting or mitigating the impact of burn injury on HIF-1α, mTORC1, mitochondria, and ER stress in mouse skeletal muscle.

In summary, burn-induced muscle metabolic derangements, such as insulin resistance and increases in lactate production and proteolysis, are associated with mitochondrial dysfunction, activation of both mTORC1 and the HIF-1α pathway, and ER stress in mice. Our data indicate that FTI-277 prevents or ameliorates these burn-induced alterations, and thereby inhibits cachectic changes in mouse skeletal muscle (Fig. 9). Based on previous studies and our data, we speculate that mTORC1, the HIF-1α pathway, mitochondrial dysfunction, and ER stress are linked to one another, forming a nexus of signaling network that contributes in concert to produce burn-induced metabolic derangements in skeletal muscle. Our data suggest that FTI-277 prevents these burn-induced metabolic derangements by inhibiting the signaling network in which mTORC1, HIF-1α, ER stress, and mitochondrial dysfunction are major players. Importantly, our study identifies protein farnesylation as a hub of the signaling network that drives burn-induced alterations in metabolism and inflammatory response and as a novel potential molecular target to prevent muscle cachexia in burn patients (Fig. 9). This ability to derail this vicious inflammatory and metabolic spiral therapeutically could have a profound impact in critical illness and burn injury.

## Materials and Methods

### Ethics statement

All experiments were carried out in accordance with the institutional guidelines and the study protocol was approved by the Institutional Animal Care and Use Committee (IACUC) at the Massachusetts General Hospital (the protocol #: 2007N000020). The animal care facility is accredited by the Association for Assessment and Accreditation of Laboratory Animal Care.

### Animals

We used male C57BL/6 mice (Jackson Laboratory, Bar Harbor, ME) at 8 weeks of age. All the mice were housed in a controlled environment (20–22 °C; 12 hours of light/dark) from seven days prior to the burn injury or sham-burn injury procedure through the end of the study. The mice were provided with standard rodent chow and water ad libitum. The pair-feeding was performed as described previously^37^. A full-thickness burn injury comprising 30% of total body surface area was produced under anesthesia with pentobarbital sodium (50 mg/kg BW, IP) by immersing the abdomen for 6 sec and both sides of the flank for 4 sec in 80 °C water as previously described^17, 37^. Sham-burned mice were immersed in lukewarm water. Starting at 2 h after burn or sham-burn, the mice were treated with FTI-277 (N-[4-[2(R)-amino-3-mercaptopropyl] amino-2-phenylbenzoyl] methionine methyl ester trifluoroacetate salt) (5 mg/kg BW/day, IP, Sigma, St. Louis, MO) or vehicle (phosphate-buffered saline [PBS]) for 3 days. To minimize animal suffering and distress after burn injury, buprenorphine (0.1 mg/kg BW, SC) was administered 30 min prior to burn and sham-burn and every 8 h for 72 h after burn or sham-burn. For resuscitation, prewarmed normal saline (0.04 ml/g BW, IP) was injected just after burn or sham-burn. Postoperatively, the mice were kept warm with heating lamps and monitored continuously until recovery of consciousness and mobility. Signs of pain and/or distress (as indicated by reduced activity, change in temperament, decreased food intake, abnormal vocalization, abnormal posture, self-mutilation of wound) and mobility of the mice were monitored every 8 h for 72 h after burn or sham-burn and once daily thereafter until the end of the study. The body weight and food intake were measured every day. None of the mice died or showed any of the signs of pain or distress, 15% or greater body weight loss compared with the initial body weight just after burn or sham-burn, or skin wound infection in this study. At 3 days after burn or sham-burn, rectus abdominis muscle was collected for biochemical analyses and transmission electron microscopy. At the end of study, the mice were euthanized by carbon dioxide asphyxiation.

### Immunoblot analysis

Immunoblotting was performed as previously described^17, 64^. Briefly, muscle tissues were pulverized under liquid nitrogen and homogenized in homogenization buffer A (50 mM HEPES pH 8.0, 150 mM NaCl, 2 mM EDTA, 7.5% SDS, 2% CHAPS, 10% glycerol, 10 mM sodium fluoride, 2 mM sodium vanadate, 1 mM PMSF, 10 mM sodium pyrophosphate, protease inhibitor cocktail). Equal amounts of protein, determined by detergent-compatible protein assay kit (Bio-Rad, Hercules, CA), were boiled for 5 min in Laemmli sample buffer, separated by SDS-PAGE, and transferred onto nitrocellulose membranes (Bio-Rad). The membranes were soaked in blocking buffer (GE Healthcare, Pittsburgh, PA) for 1 h and then incubated overnight at 4 °C with anti-glyceraldehyde 3-phosphate dehydrogenase (GAPDH) (Trevigen, Gaithersburg, MD, #2275-PC-1) (dilution 1:25,000), anti-HIF-1α (1:2,000) (Cayman Chemical, Ann Abor, MI, #10006421), anti-PKM2 (#3198) (1:5,000), anti-PKM1 (#7067) (1:25,000), anti-phospho-mTOR (Ser 2448) (#5536) (1:2,000), anti-mTOR (#2983) (1:5,000), anti-phospho-p70S6K (Ser 371) (#9208) (1:2,000), anti-p70S6K (#2708) (1:5,000), anti-phospho-S6 (Ser 235/236) (#2211) (1:2,000), anti-S6 (#2217) (1:5,000), anti-phospho-4EBP1 (Thr 37/46) (#2855) (1:25,000), anti-4EBP1 (#9644) (1:25,000), anti-phospho-p38 MAPK (Thr 180/Tyr 182) (#9211) (1:5,000), anti-p38 MAPK (#9212) (1:5,000), anti-phospho-p44/42 MAPK (Erk1/2) (Thr 202/Tyr 204) (#4370) (1:5,000), anti-p44/42 MAPK (Erk1/2) (#4695) (1:5,000), anti-phospho-SAPK/JNK (Thr 183/Tyr 185) (#4668) (1:5,000), anti-SAPK/JNK (#9258) (1:5,000) (Cell Signaling Technology, Danvers, MA), anti-phospho-IRE1 (Ser 724) (Novus Biological, Littleton, CO, #NB100-2323) (1:2,000) and anti-IRE1 (#ab37073) (1:5,000), anti-GRP78 BiP (ab21685) (1:5,000), anti-Neutrophil Elastase (ab68672) (1:5,000) (Abcam, Cambridge, MA) and anti-Actin (A5060) (1:25,000) (Sigma) followed by incubation with secondary antibody (antibody recognizing rabbit or mouse IgG) conjugated to horseradish peroxidase for 1 h at room temperature (1:25,000 or 1:50,000). Antigen-antibody complexes were detected by enhanced chemiluminescence reagent (Lumigen, Southfield, MI). The immunoblots were scanned using the HP Scanjet 4850 (Hewlett-Packard, Palo Alto, CA). Densitometric analysis of the results was carried out using NIH ImageJ software (ver. 1.62).

### Isolation of total RNA and quantitative reverse transcription polymerase chain reaction (RT-PCR)

Total RNA was isolated with TRIzol reagent (Life Technologies, Grand Island, NY) from skeletal muscle at 3 days after burn or sham-burn injury. The first-strand cDNA was synthesized from 1 μg of total RNA using the High Capacity cDNA Reverse Transcription Kit (Applied Biosystems, Waltham, MA). RT-PCR was performed using 10 ng of cDNA and TaqMan probes (Applied Biosystems) for HIF-1α (Mm00468869_m1), Glut1 (Mm00441480_m1), PDK1 (Mm00554300_m1), and GAPDH (Mm99999915_g1) using Mastercycler (Eppendorf, Westbury, NY). mRNA levels were normalized to that of GAPDH.

### Measurement of mitochondrial respiration

The Seahorse XFp Extracellular Flux Analyzer (Seahorse Bioscience, North Billerica, MA) was used to measure the mitochondrial oxygen consumption rate (OCR) as an indicator of mitochondrial respiration. For these measurements, we used fresh muscle as described previously^65^. Briefly, to isolate the mitochondrial fraction, immediately after the muscles were excised under anesthesia, they were minced in ~10 volumes of cold MSHE + BSA buffer (70 mM sucrose, 210 mM mannitol, 5 mM HEPES, 1 mM EGTA and 0.5% fatty acid-free BSA, pH 7.2) at 4 °C and all subsequent steps of the preparation were performed on ice. The tissues were homogenized using a drill-driven Teflon dounce homogenizer for 10 strokes. Homogenates were centrifuged at 800 g for 10 minutes at 4 °C. Following the centrifugation, the fat/lipid layer was carefully aspirated, and the remaining supernatant was decanted through 2 layers of cheesecloth into a separate tube and centrifuged at 8,000 g for 10 minutes at 4 °C. After removing the light layer, the final pellets were resuspended in a minimal volume of MSHE + BSA buffer. Total protein concentrations in the mitochondrial fraction were determined using the Bradford Assay reagent (Bio-Rad) and the concentration was adjusted to 0.1 mg/ml. Then, 25 μl (2.5 μg) of isolated mitochondria was applied into each well and centrifuged at 2,000 g for 20 min at 4 °C. After the centrifugation, 155 µl of pre-warmed (37°) 1x MAS buffer (70 mM sucrose, 220 mM mannitol, 10 mM KH_2_PO_4_, 5 mM MgCl_2_, 2 mM HEPES, 1 mM EGTA and 0.2% fatty acid-free BSA, pH 7.2) + substrates were added to each well and incubated at 37 °C in a non-CO_2_ incubator. The plate was transferred to the XFp analyzer after confirming consistent adherence of the mitochondria under a microscope. Two experimental designs were used for this experiment. In the first design, mitochondrial OCR was sequentially measured in the following states; (i) basal respiration in the presence of substrates (2 mM of glutamine, 5 mM of pyruvate, and 2.5 mM malate), (ii) phosphorylating respiration induced by 4 mM of adenosine diphosphate (ADP), (iii) in the presence of the ATP synthase inhibitor, 2.5 μg/ml oligomycin, and (iv) maximal respiration induced by 5 μM of carbonyl cyanide 4-(trifluoromethoxy) phenylhydrazone (FCCP); a mitochondrial uncoupler, with 0.5 mM of reduced form of nicotinamide adenine dinucleotide (NADH) and 100 μM of decylubiquinone. ADP-stimulated OCR and FCCP-stimulated OCR were assessed by the change in OCR between pre- and post-injection of ADP and FCCP, respectively. The second type of experiment examined sequential electron flow through different complexes of the electron transport chain. This experiment began with the mitochondria utilizing complex I respiration in an uncoupled state (10 mM pyruvate, 2 mM malate, and 4 μM FCCP). The following compounds (final concentrations) were then added sequentially: rotenone (2 mM), succinate (10 mM), antimycin A (4 mM), and ascorbate/ N, N, N9, N9-Tetramethylp-phenylenediamine (TMPD) (1 mM and 100 mM, respectively). Because oxidation of pyruvate/malate is mediated via complex I, injection of rotenone inhibits complex I-dependent electron transport. Injection of succinate allows the mitochondria to respire via complex II, and OCR values increase. Electron flow was then inhibited at complex III by antimycin A. Finally, addition of ascorbate and TMPD (which act as electron donors to cytochrome C/complex IV) elicited an increase in the OCR. Complex I-, complex II-, and complex IV-dependent OCRs were assessed by the OCR change between pre- and post-injection of rotenone, succinate, and ascorbate/TMPD, respectively.

### Transmission electron microscopy

Tissue samples, which were collected from one mouse per each of the four groups, were immediately fixed in a 2.5% paraformaldehyde/2.5% glutaraldehyde solution in 0.1 M sodium cacodylate buffer (pH 7.4) (Electron Microscopy Sciences, Hatfield, PA), followed by osmication, uranyl acetate staining, and dehydration in alcohols. The samples were then embedded in Taab 812 Resin (Marivac Ltd., Nova Scotia, Canada). Sample blocks were cut (80 nm sections) with a Leica ultracut microtome, picked up on 100 mesh formvar/carbon-coated Cu grids (Electron Microscopy Sciences), stained with 0.2% lead citrate, and viewed and imaged under the Philips Technai BioTwin Spirit Electron Microscope (FEI, Hillsboro, OR) at the Harvard Medical School Electron Microscopy facility. The number and size of the intermyofibrillar mitochondria were evaluated by tracing by the outline of the mitochondria at 6,800× magnification using ImageJ software^66^. The mitochondrial density is expressed as the percent of total myofiber area^66^. An average of 83 mitochondria were traced per animal. To evaluate partial loss of cristae structure, the length of cristae was measured at 18,500× magnification and normalized to the mitochondrial area.

### Blue native PAGE

Two-dimensional blue native PAGE was performed as described previously^67^. Briefly, skeletal muscle, which was collected from one mouse per each of the four groups, was homogenized with a glass Teflon homogenizer in buffer containing 10 mM HEPES-KOH (pH 7.4), 0.22 M mannitol, 0.07 M sucrose, and 0.1 mM EDTA. The extracts were then centrifuged at 500 g and the mitochondrial fraction was precipitated by further centrifugation at 10,000 g. The mitochondria (100 μg mitochondrial protein) were suspended in 10 μl of a buffer containing 50 mM Bis-Tris and 1 M 6-aminocaproic acid. Digitonin was added to solubilize the mitochondria. After a 30-min incubation at 4 °C, the solubilized proteins were obtained from the supernatant fraction by centrifugation at 22,000 g. The solubilized proteins were supplemented with 1 μl of sample buffer (5% Coomassie brilliant blue G-250 in 0.5 M 6-aminocaproic acid). A stacking gel (4%) and separating gels with a stepwise gradient of 8, 9, 10 and 11% were cast and electrophoresed. The second-dimension SDS-PAGE and immunoblotting were performed according to standard protocols, and the blot was probed with anti-NDUFA9 (#459100), anti-succinate dehydrogenase complex, subunit A (SDHA) (#459200) (Invitrogen, Waltham, MA), anti-UQCRC2 (#ab14745), and anti-RISP antibodies (#ab14746) (Abcam). Signal intensities of the blots were evaluated by ImageJ.

### Statistical analysis

The data were compared with two-way ANOVA followed by Tukey multiple comparison test. A value of p < 0.05 was considered statistically significant. All values are expressed as means ± SEM.

## Electronic supplementary material

Supplementary Information
