# Supplementary material for: Burn-induced muscle metabolic derangements and mitochondrial dysfunction are associated with activation of HIF-1α and mTORC1: Role of protein farnesylation
Source: Sci Rep. 2017 Jul 26;7:6618. doi: 10.1038/s41598-017-07011-3 (PMC5529411; doi:10.1038/s41598-017-07011-3)
Supplement: Supplementary file 1 — Supplementary Information [file 41598_2017_7011_MOESM1_ESM.pdf]

Supplementary Information

Title: Burn-induced muscle metabolic derangements and mitochondrial dysfunction are associated with activation of HIF-1α and mTORC1: Role of protein farnesylation

Author Names: Harumasa Nakazawa[1,2,3], Kazuhiro Ikeda[4], Shohei Shinozaki[1,2,5,], Masayuki Kobayashi[1,2], Yuichi Ikegami[1], Ming Fu[1,2], Tomoyuki Nakamura[1,2], Shingo Yasuhara[1,2], Yong-Ming Yu[2,6], J.A.Jeevendra Martyn[1,2], Ronald G. Tompkins[2,6], Kentaro Shimokado[5], Tomoko Yorozu[3], Hideki Ito[7], Satoshi Inoue[4,7], Masao Kaneki[1,2]*

[1]Department of Anesthesia, Critical Care and Pain Medicine, Massachusetts General Hospital, Harvard Medical School, Charlestown, MA 02129, USA

[2]Shriners Hospitals for Children, Boston, MA 02114, USA

[3]Department of Anesthesiology, Kyorin University School of Medicine, Tokyo, Japan

[4]Division of Gene Regulation and Signal Transduction, Research Center for Genomic Medicine, Saitama Medical University, Saitama, Japan

[5]Department of Geriatrics and Vascular Medicine, Tokyo Medical and Dental University Graduate School, Tokyo, Japan

[6]Department of Surgery, Massachusetts General Hospital, Harvard Medical School, Boston, MA 02114, USA

[7]Tokyo Metropolitan Institute of Gerontology, Tokyo, Japan

* Corresponding author. Masao Kaneki, M.D., Ph.D.

E-mail: mkaneki@helix.mgh.harvard.edu

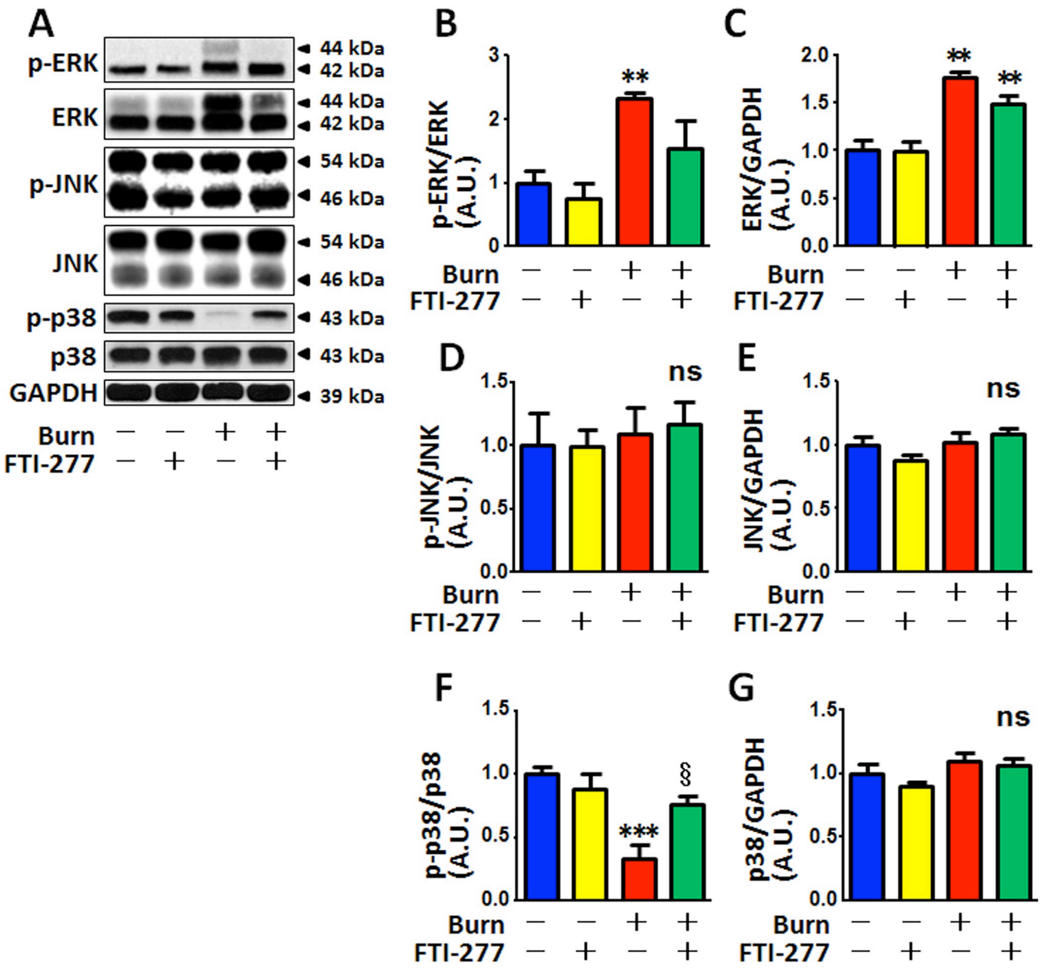

Supplementary Figure 1. Effects of burn injury on phosphorylation of MAPKs. At 3 days after burn or sham-burn, phosphorylation of ERK, JNK and p38 MAPK was evaluated by immunoblotting in mouse skeletal muscle. When treated with vehicle alone, burn injury increased phosphorylation of ERK compared with sham-burn (A, B). Phosphorylation of ERK in FTI-277-treated burned mice appeared to be greater than sham-burned mice and lower than vehicle-treated burned mice, but there were no statistically significant differences. Burn injury increased ERK expression compared with sham-burn regardless of FTI-277 treatment (A, C).

Neither burn injury nor FTI-277 altered phosphorylation and protein expression of JNK (A, D, E). On the other hand, burn injury decreased phosphorylation of p38 MAPK compared with sham-burn, which was inhibited by FTI-277 (A, F). p38 MAPK expression was not significantly altered by burn injury or FTI-277 (A, G). These results indicate that the effects of burn injury on phosphorylation of MAPKs varied between ERK, JNK, and p38 MAPK. **P<0.01, ***P<0.001 vs. sham-burn groups, §P<0.05 vs. vehicle-treated burn group, ns: not significant. n=6 per group.

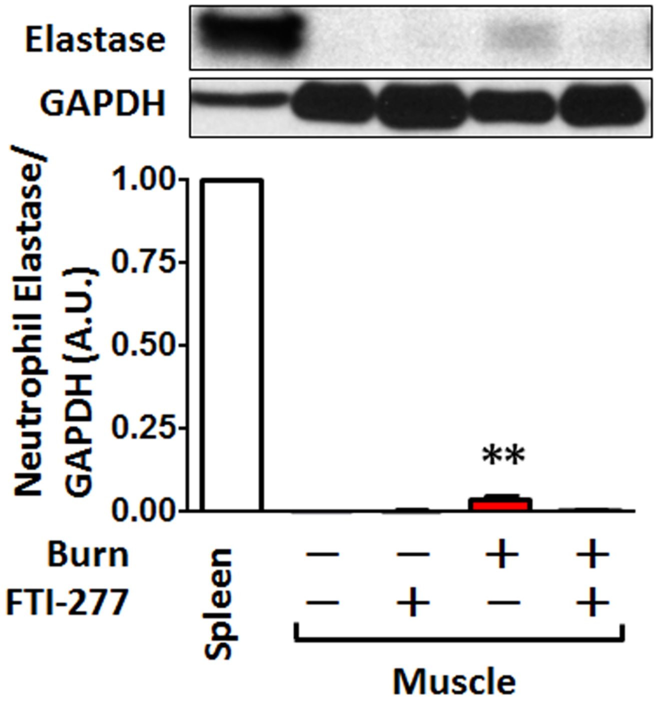

Supplementary Figure 2. Neutrophil elastase expression in mouse skeletal muscle tissue after burn injury. To assess the degree of neutrophil infiltration into skeletal muscle tissue, we evaluated neutrophil elastase content in the homogenates of skeletal muscle tissue at 3 days post-burn or sham-burn (n=3 per group). The homogenates of spleen from naïve mice (n=2) were used as a control. In sham-burned mice, neutrophil elastase was not detectable in skeletal muscle tissue. In contrast, burn injury increased neutrophil elastase content in skeletal muscle tissue, which was inhibited by FTI-277. The average level of neutrophil elastase in skeletal muscle of vehicle-treated burned mice was 3.8% of the levels found in spleen. **$P<0.01$ vs. sham-burn groups and FTI-277-treated burn group.
